# Association of Circulating Tumor DNA With Brain Metastases: A Systematic Review and Meta‐Analysis

**DOI:** 10.1002/cns.70965

**Published:** 2026-06-03

**Authors:** Shaobo Yang, Zongheng Zhang, Hao Dong, Xin Yu, Fangdi Zhu, Shun Yang

**Affiliations:** ^1^ Department of Neurosurgery Changde Hospital, Xiangya School of Medicine, Central South University (The First People's Hospital of Changde City) Changde Hunan China

**Keywords:** brain metastases, ctDNA, meta‐analysis

## Abstract

**Background:**

Brain metastases are a major contributor to morbidity and mortality in patients with advanced solid tumors; however, early detection and accurate prognostic assessment remain significant clinical challenges. ctDNA is a minimally invasive biomarker that allows real‐time monitoring of tumor behavior and provides information on systemic tumor burden and molecular diversity. This study aimed to systematically evaluate the diagnostic and prognostic value of ctDNA in brain metastases, with particular emphasis on the complementary roles of plasma and CSF ctDNA.

**Methods:**

PubMed, Embase, Cochrane Library, Web of Science, and ScienceDirect were systematically searched up to January 2025. Pooled ORs, SMDs, and HRs with 95% CIs were calculated using fixed‐effects or random‐effects models according to heterogeneity.

**Results:**

Fifteen studies (*N* = 1763) were included. ctDNA positivity was significantly associated with increased risk of brain metastases (OR = 1.67, 95% CI: 1.24–2.26, *p* = 0.001). Subgroup analysis showed that NGS had notably higher predictive performance (OR = 5.50, 95% CI: 1.92–15.76, *p* = 0.002). Plasma ctDNA levels were significantly higher in patients with brain metastases (SMD = 0.51, 95% CI: 0.18–0.84, *p* = 0.002). Notably, CSF ctDNA positivity was strongly associated with worse overall survival (HR = 2.75, 95% CI: 1.75–4.32, *p* < 0.001).

**Conclusion:**

Plasma ctDNA serves as a non‐invasive biomarker for systemic metastatic risk, whereas CSF ctDNA provides superior prognostic value for intracranial disease. Using these biomarkers together in a clinical workflow could enhance risk assessment and guide treatment decisions in patients with brain metastases.

## Graphical abstract

This meta‐analysis of 1763 patients confirms ctDNA as a key biomarker for brain metastases. While plasma ctDNA effectively indicates metastatic risk and tumor burden, CSF ctDNA offers superior prognostic value for survival. Integrating both biomarkers into clinical workflows optimizes risk assessment and guides precision treatment for intracranial disease.

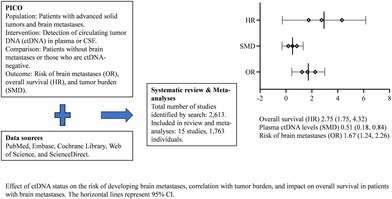

## 1 Introduction

Brain metastases remain a life‐threatening complication in patients with malignant tumors. Epidemiological studies indicate that approximately 10%–20% of adult cancer patients develop brain metastases during their disease course, with poor prognosis and a 1‐year survival rate below 60% [1]. Despite significant advancements in imaging technologies and localized treatment modalities in recent years, the early diagnosis and precise prognostic assessment of brain metastases remain major clinical challenges [2]. Molecular characteristics of brain metastases often differ substantially from those of primary tumors, a divergence further supported by genomic studies. These differences may influence treatment response and limit the utility of traditional tissue biopsies for therapeutic decision‐making [3, 4]. Collectively, these limitations underscore a critical unmet need for noninvasive biomarkers capable of dynamic monitoring and capturing both systemic and intracranial disease evolution.

Circulating tumor DNA (ctDNA), an emerging liquid biopsy technology, has demonstrated substantial potential in the diagnosis and therapeutic monitoring of metastatic cancers [5]. ctDNA comprises tumor‐derived DNA fragments released into the circulation through apoptosis, necrosis, or active secretion, constituting the tumor‐specific component of cell‐free DNA (cfDNA) [6, 7, 8]. Given the short half‐life of cfDNA (less than 1 h), ctDNA levels reflect real‐time tumor burden, with studies suggesting its superior sensitivity compared to radiographic imaging [9, 10, 11]. Accumulating evidence highlights the value of ctDNA in early cancer detection, treatment response evaluation, minimal residual disease (MRD) monitoring, and prognostic prediction [12, 13]. Importantly, ctDNA detection can signal disease recurrence months earlier than imaging, offering a critical window for clinical intervention [14, 15]. However, peripheral blood ctDNA may inadequately capture molecular features of central nervous system (CNS) metastases [16], whereas cerebrospinal fluid (CSF) ctDNA may more directly reflect genetic alterations in brain metastases [17]. Furthermore, existing studies predominantly focus on ctDNA in extracranial metastases, with fragmented evidence and methodological heterogeneity (e.g., small sample sizes, diverse detection platforms) limiting conclusive insights into its role in brain metastasis management.

The relationship between ctDNA and brain metastases is complex but offers potential diagnostic and therapeutic insights. In a diagnostic study of leptomeningeal melanoma metastasis (LMM), CSF ctDNA analysis targeting BRAF V600 and NRASQ61 mutations demonstrated 81.8% sensitivity and 100% specificity. This approach also resolved discordant imaging and cytology findings using a rapid 110‐min assay, thereby facilitating clinical decisions [18]. Integrating ctDNA analysis into clinical workflows provides actionable insights for patient management. For instance, longitudinal monitoring of ctDNA mutations in breast cancer enables dynamic assessment of tumor genomic profiles, guiding therapeutic adjustments [19]. Clinical studies have established correlations between ctDNA levels, tumor burden, treatment response, and prognosis [20]. However, consensus remains elusive regarding ctDNA's association with brain metastasis risk and survival outcomes. Early observational data suggest that persistent detection of tumor‐associated mutations in ctDNA may outperform traditional clinicopathological risk factors in prognostic stratification [21].

This systematic review and meta‐analysis synthesizes high‐quality evidence to address three key research questions: (1) Is ctDNA significantly associated with the risk of developing brain metastases? (2) Can ctDNA serve as a reliable prognostic biomarker for survival outcomes in patients with brain metastases? (3) Does the predictive performance of ctDNA vary across primary tumor types and detection methods? This study addresses these questions to provide evidence supporting the clinical use of ctDNA in managing brain metastases and to inform precision treatment strategies.

## 2 Materials and Methods

### 2.1 Search Strategy

We searched PubMed, Web of Science, Cochrane Library, Embase, and ScienceDirect from their inception to January 2025, without restricting publication type or language. Key search terms included “Circulating Tumor DNA” [MeSH], “brain metastasis” [MeSH], and their derivatives. We also manually checked reference lists from included studies and relevant reviews to find additional studies.

### 2.2 Study Selection

Two independent reviewers screened titles, abstracts, and full texts for eligibility. Disagreements were resolved through consensus or consultation with a third reviewer. Inclusion criteria were: (1) Peer‐reviewed original articles published in English or with accessible English abstracts; (2) Studies involving patients with confirmed brain metastases; (3) Investigations of associations between ctDNA and brain metastasis development or outcomes; (4) Reported outcomes including overall survival (OS), progression‐free survival (PFS), or brain metastasis‐related risk metrics; (5) Sufficient data to calculate effect sizes (e.g., hazard ratios (HRs), odds ratios (ORs), standardized mean differences (SMDs)) or associations.

Exclusion criteria included: (1) Non‐original research (reviews, case reports, editorials); (2) Studies lacking explicit brain metastasis data or ctDNA brain metastasis correlations; (3) Non‐human or in vitro studies; (4) Insufficient statistical data (e.g., missing standard deviations, confidence intervals (CIs), or precise *p*‐values); (5) Duplicate patient cohorts (only the most comprehensive or recent publication was retained).

### 2.3 Data Extraction

Two researchers independently extracted data using a standardized form, including:

Study characteristics: first author, publication year, country, sample size, target mutations, tumor subtypes, study design, sample type (plasma/CSF), and Newcastle‐Ottawa Scale (NOS) score [22].

Outcome data: number of ctDNA‐positive brain metastasis cases vs. controls, means ± standard deviations, and HRs with 95% CIs for OS/PFS. Disagreements were resolved by discussion among the reviewers.

### 2.4 Quality Assessment

Study quality was independently evaluated by two reviewers (Y.S.B. and Y.S.) using the NOS score, which assigns scores across three domains: Selection (4 points: representativeness of cohorts, exposure/outcome ascertainment); Comparability (2 points: adjustment for confounders); Outcome (3 points: outcome assessment and follow‐up adequacy). Studies with NOS scores ≥ 6 were classified as high quality.

### 2.5 Data Synthesis and Analysis

The predictive ability of ctDNA in determining the risk of brain metastasis was evaluated using ORs and SMDs with 95% CIs. To evaluate the prognostic significance of ctDNA in brain metastasis, HRs with 95% CIs were used for meta‐analysis.

The heterogeneity between studies was assessed using Cochran's *Q* test and Higgins *I* ^2^ statistic. When significant heterogeneity was detected (*I* ^2^ > 50% or *p* < 0.1), a random effects model was used; otherwise, a fixed effects model was used. Subgroup analysis and meta‐regression analysis stratified by different factors were performed to explore the sources of heterogeneity. Sensitivity analysis was performed to evaluate the stability of the combined results. Publication bias was assessed using Begg's test and Egger's test. Statistical significance was defined as a two‐tailed *p* value < 0.05. Meta‐analysis was performed using STATA 14.0.

## 3 Results

### 3.1 Characteristics and Quality Assessment of Included Studies

**FIGURE 1:**
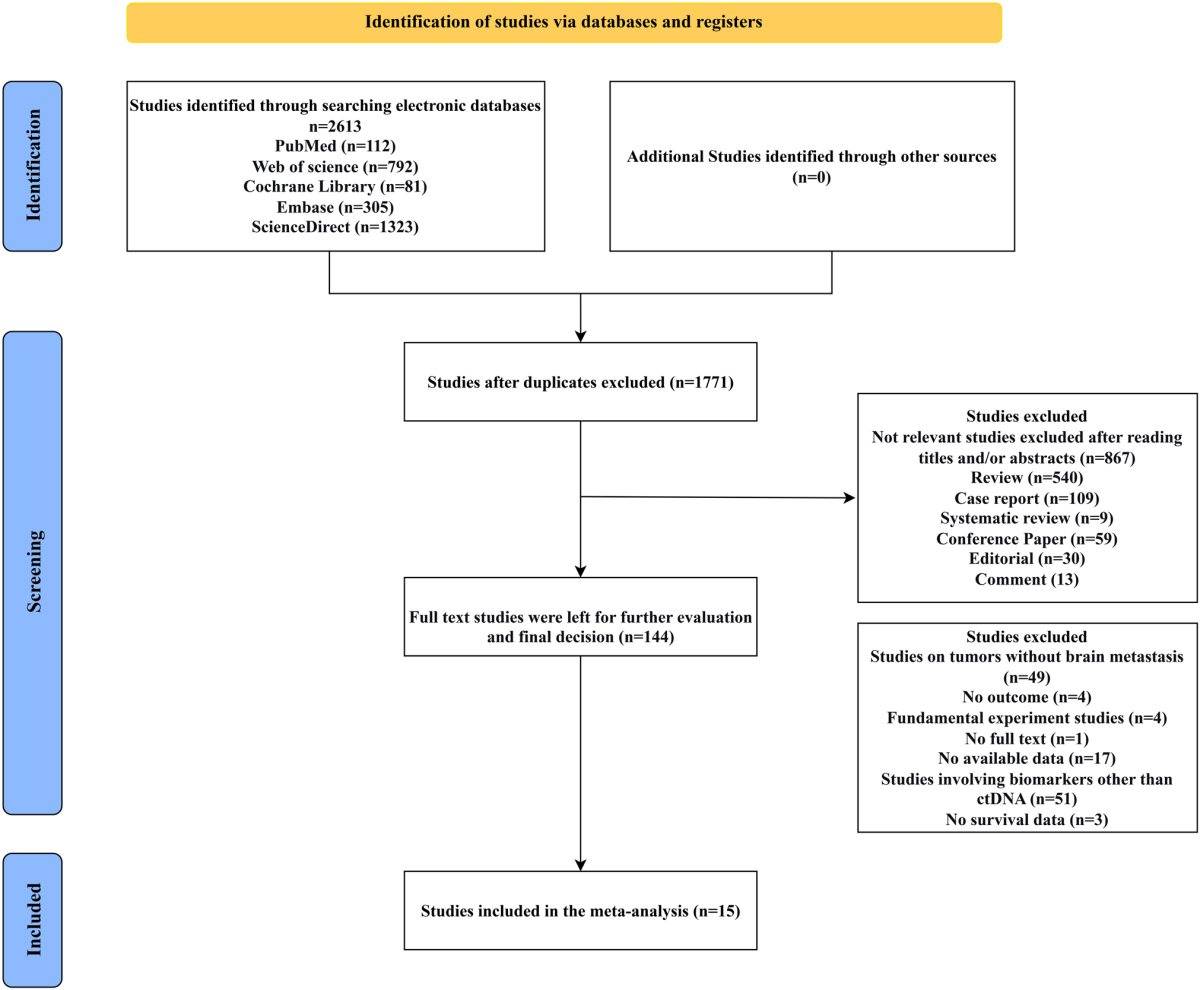
PRISMA flow diagram of the study selection process.

The literature screening process is summarized in Figure 1. A total of 1771 studies were initially identified through electronic databases and manual searches. After removing duplicates, 867 studies were excluded based on title/abstract screening. Subsequent full‐text reviews excluded 540 reviews, 109 case reports, 9 systematic reviews, 59 conference papers, 30 editorials, and 13 commentaries. Of the 144 studies assessed for eligibility, 129 were excluded due to: absence of brain metastasis data (*n* = 49), lack of relevant outcomes (*n* = 4), preclinical focus (*n* = 4), unavailability of full texts (*n* = 1), Studies with insufficient statistical data (e.g., missing standard deviations, confidence intervals, or precise *p*‐values) were excluded to ensure reliable quantitative synthesis (*n* = 17), non‐ctDNA biomarkers (*n* = 51), or missing survival metrics (*n* = 3). Ultimately, 15 studies met inclusion criteria for meta‐analysis [12, 23, 24, 25, 26, 27, 28, 29, 30, 31, 32, 33, 34, 35, 36].

Table 1 summarizes the characteristics and quality scores of the 15 included studies, published between 2015 and 2024, encompassing 1763 patients. Plasma and CSF samples were predominantly analyzed, with ctDNA detection methods including droplet digital PCR (ddPCR) and next‐generation sequencing (NGS). All studies achieved NOS scores ≥ 6, indicating satisfactory methodological quality.

**TABLE 1:** Characteristics of the included meta‐analytic studies.

Study ID	Year	Country	Sample size	Target mutation	Method of ctDNA analysis	Subtype	Design	Sample	NOS
Chang et al.	2015	USA	73	BRAF and NRAS	ddPCR	Metastatic melanoma	Prospective	Plasma	7
Dayimu et al.	2024	UK	79	BRAF	ddPCR	Advanced melanoma	Prospective	Plasma	8
Fietz et al.	2024	Germany	42	SHOX2	PCR	Melanoma	Prospective	Plasma	7
Fitzpatrick et al.	2022	UK	30	/	ddPCR	Breast Cancer	Prospective	CSF	7
Kim et al.	2022	Korea	311	EGFR	RT‐PCR	NSCLC	Retrospective	Plasma	8
Li et al.	2022	China	92	EGFR, TP53, and KRAS	NGS	NSCLC	Prospective	CSF	7
Liu et al.	2021	China	79	EGFR	ddPCR	Lung adenocarcinoma	Retrospective	CSF	8
Mack et al.	2022	USA	106	EGFR	NGS	NSCLC	Prospective	Plasma	7
Moiseenko et al.	2022	Russia	99	EGFR	/	NSCLC	Retrospective	Plasma	7
Pentsova et al.	2016	USA	53	EGFR	PCR	NSCLC Breast Melanoma Bladder cancer Ovarian	Retrospective	CSF	6
Xie et al.	2020	China	146	FGFR	NGS	Breast cancer	Prospective	Plasma	7
Zhu et al.	2017	China	113	EGFR	ddPCR	NSCLC	Prospective	Plasma	6
Zugazagoitia et al.	2019	Spain	93	EGFR, ALK and ROS1	NGS	Advanced‐stage lung adenocarcinomas	Prospective	Plasma	7
Bachet et al.	2018	France	412	RAS	NGS and ddPCR	Colorectal cancer	Prospective	Plasma	6
Iwama et al.	2017	Japan	35	EGFR	dPCR and NGS	Lung adenocarcinoma	Prospective	Tissue and plasma	8

#### 3.1.1 Association Between ctDNA and Brain Metastases Risk

**FIGURE 2:**
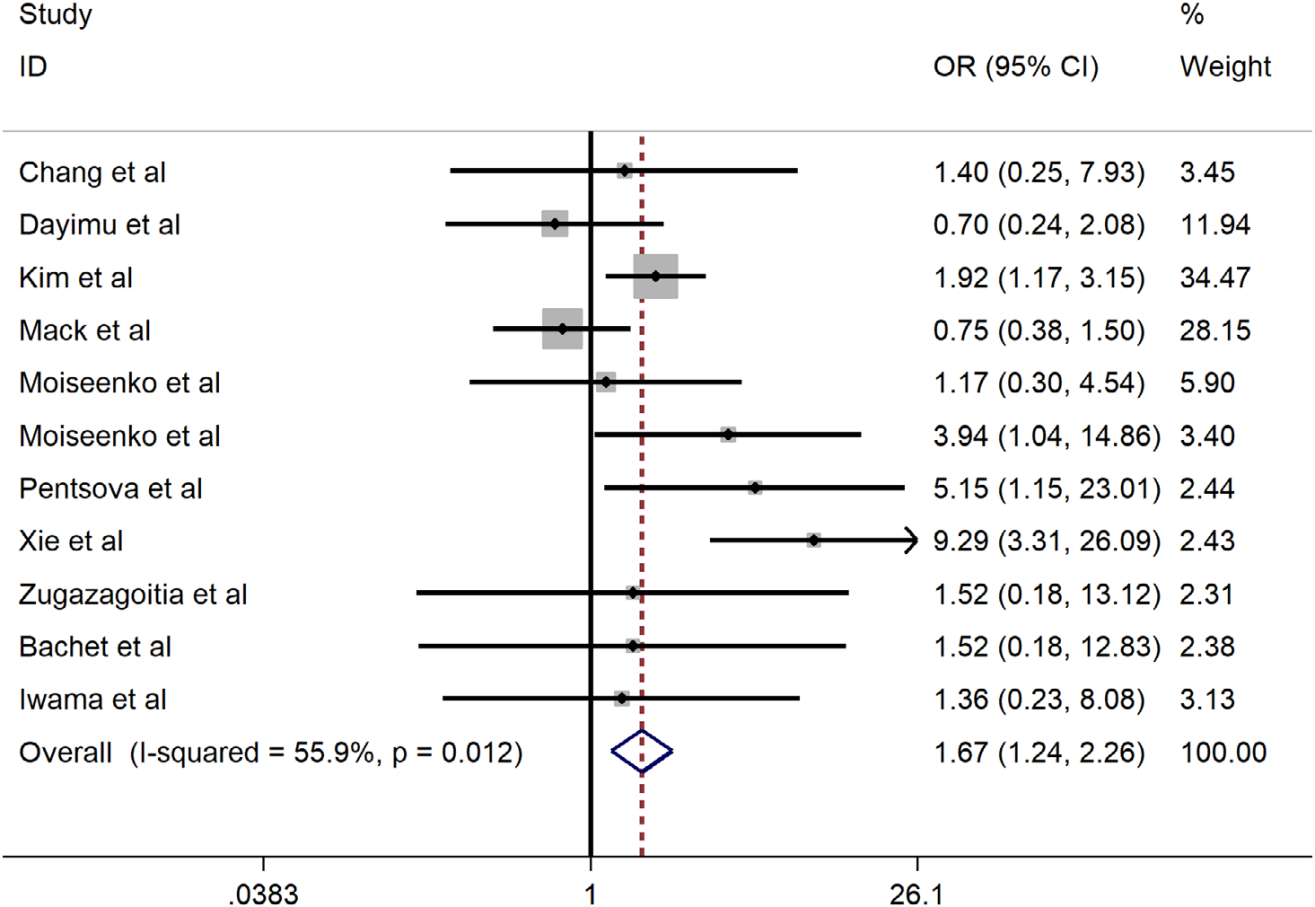
Forest plot of the association between ctDNA and the risk of brain metastasis (11 studies, *N* = 1407). A fixed‐effects model was applied.

Eleven studies evaluated the predictive role of ctDNA for brain metastasis. The pooled OR was 1.67 (95% CI: 1.24–2.26; *p* = 0.001; Figure 2), indicating a significant association.

The results of the subgroup analyses are presented in Table 2:

**TABLE 2:** Subgroup analysis of the association between ctDNA and the risk of brain metastasis.

Subgroup analysis		Number of studies	OR (95% CI)	Heterogeneity		*p*
				*I* ^2^ (%)	*p*	
Primary tumor	Melanoma	2	0.86 (0.35–2.11)	0.0%	0.507	0.737
	NSCLC	3	1.50 (1.04–2.16)	56.9%	0.073	0.031
	Lung adenocarcinoma	2	1.43 (0.36–5.62)	0.0%	0.939	0.608
Target mutation	EGFR Mutation	6	1.73 (1.24–2.44)	72%	0.003	0.001
	Others	4	2.32 (0.93–5.78)	0.0%	0.634	0.071
Detection methods	ddPCR	4	1.76 (1.16–2.65)	39%	0.178	0.007
	NGS	2	5.50 (1.92–15.76)	57.6%	0.125	0.002

Primary tumor type: Non‐small cell lung cancer (NSCLC): OR = 1.50 (95% CI: 1.04–2.16; *p* = 0.031); Melanoma: OR = 0.86 (95% CI: 0.35–2.11; *p* = 0.737); Lung adenocarcinoma: OR = 1.43 (95% CI: 0.36–5.62; *p* = 0.608).

Mutation profile: EGFR mutations: OR = 1.73 (95% CI: 1.24–2.44; *p* = 0.001); Non‐EGFR alterations: OR = 2.32 (95% CI: 0.93–5.78; *p* = 0.071).

Detection method: ddPCR: OR = 1.76 (95% CI: 1.16–2.65; *p* = 0.007); NGS: OR = 5.50 (95% CI: 1.92–15.76; *p* = 0.002).

#### 3.1.2 Prognostic Value of ctDNA in Brain Metastasis

**FIGURE 3:**
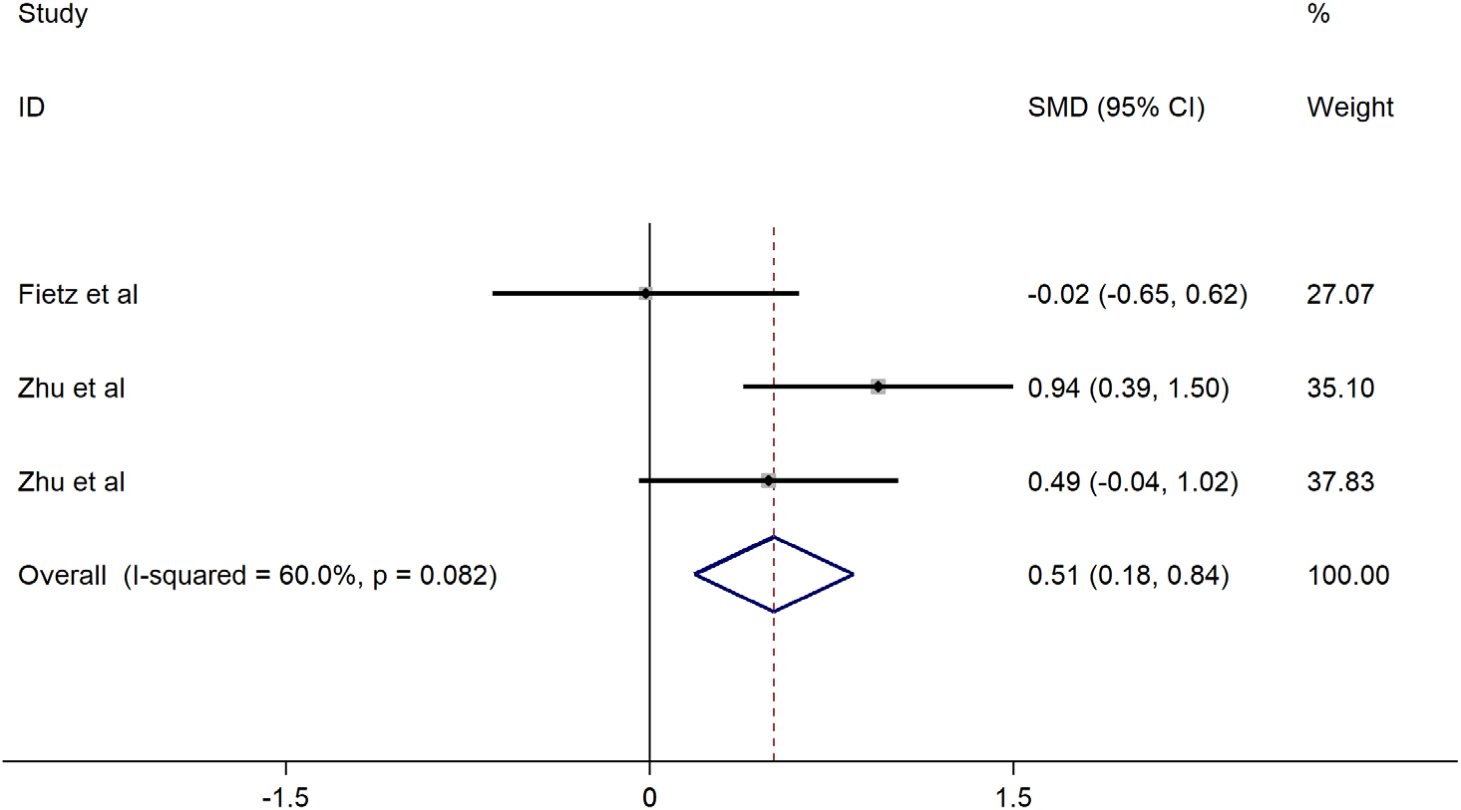
Forest plot of the association between ctDNA levels and brain metastasis (3 studies, *N* = 155). A fixed‐effects model was applied.

Three studies showed that plasma ctDNA levels were significantly elevated in patients with brain metastases versus non‐metastatic controls (SMD = 0.51; 95% CI: 0.18–0.84; *p* = 0.002; Figure 3).

#### 3.1.3 CSF ctDNA as an Independent Predictor of Mortality

**FIGURE 4:**
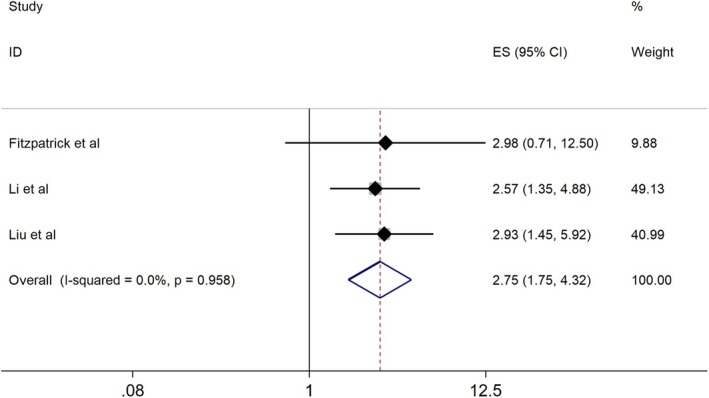
Forest plot of HRs from multivariate analyses of OS associated with ctDNA in patients with brain metastases (3 studies, *N* = 201). A random‐effects model was applied.

A pooled analysis of three studies revealed a strong association between CSF ctDNA detection and reduced OS. The HR for mortality was 2.75 (95% CI: 1.75–4.32; *p* < 0.001; Figure 4), establishing CSF ctDNA as an independent predictor of poor prognosis in brain metastasis patients.

### 3.2 Sensitivity Analysis

**FIGURE 5:**
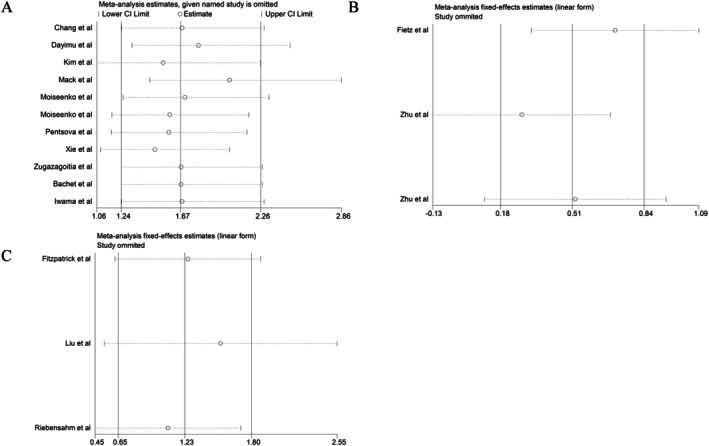
Sensitivity analysis. (A) Sensitivity analysis of the OR value of ctDNA and brain metastasis risk. (B) Sensitivity analysis of the SMD value of ctDNA and brain metastasis. (C) Sensitivity analysis of the HR value of ctDNA and brain metastasis.

Sensitivity analyses confirmed the robustness and stability of the meta‐analytic findings (Figure 5A–C). Sequentially excluding individual studies did not substantially change the pooled OR, SMD, or HR, showing that no single study had an undue influence. This consistency underscores the reliability of the conclusions across heterogeneous methodologies and patient cohorts.

### 3.3 Publication Bias

Publication bias was rigorously assessed using Begg's rank correlation test and Egger's linear regression test. For studies evaluating ctDNA's association with brain metastasis risk (*n* = 11), both tests revealed no significant bias: Begg's test: *z* = 0.31, *p* = 0.755; Egger's test: *t* = 0.39, *p* = 0.706.

For studies examining the association between ctDNA levels and brain metastasis occurrence (*n* = 3), Begg's test yielded *z* = −0.52, *p* = 0.602, and Egger's test yielded *t* = −1.05, *p* = 0.484. Similarly, for studies investigating ctDNA's prognostic value in brain metastases survival (*n* = 3), Begg's test yielded *z* = 0.52, *p* = 0.602, and Egger's test yielded *t* = 0.59, *p* = 0.659.

**FIGURE 6:**
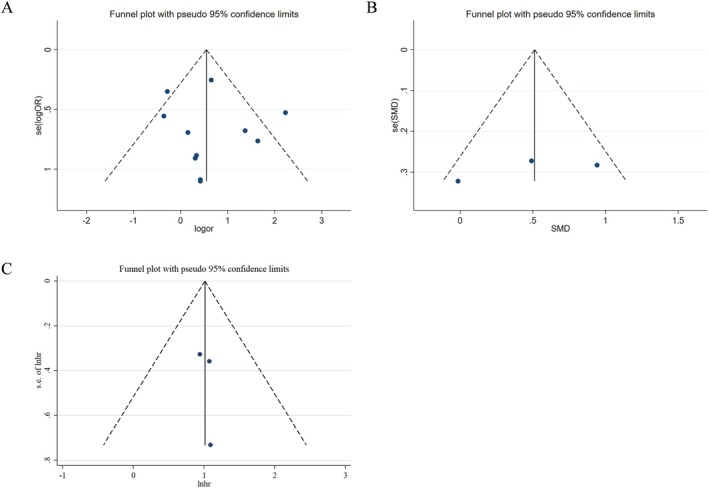
Funnel plot. (A) Funnel plot of ctDNA and the OR value of brain metastasis risk. (B) Funnel plot of ctDNA level and SMD value of brain metastasis. (C) Funnel plot of ctDNA and the HR value of brain metastasis.

All *p* values were above 0.05, suggesting no detectable publication bias. Visual inspection of funnel plots further supported these findings, demonstrating symmetrical distributions of effect sizes across studies (Figure 6A–C). This symmetry suggests minimal risk of small‐study effects or selective reporting, reinforcing the validity of the synthesized evidence.

## 4 Discussion

Brain metastasis remains a major clinical challenge despite advances in multimodal therapies, largely due to the lack of effective tools for early detection and accurate prognostic stratification [37]. In this context, ctDNA has emerged as a promising biomarker for dynamic disease monitoring. Importantly, our findings support an integrated plasma and CSF ctDNA analysis strategy, in which plasma and CSF ctDNA provide complementary insights into systemic and intracranial disease, respectively.

ctDNA is increasingly recognized as a minimally invasive biomarker for detecting micrometastatic disease and monitoring tumor dynamics [38]. Notably, it has demonstrated higher sensitivity than imaging modalities for identifying MRD and has shown value in recurrence risk stratification and treatment monitoring [39].

Our meta‐analysis demonstrates that plasma ctDNA positivity is significantly associated with an increased risk of brain metastases, and that ctDNA levels are elevated in affected patients, suggesting its potential utility as an indicator of systemic tumor burden and metastatic risk. This observation is consistent with prior evidence demonstrating a strong correlation between ctDNA levels and overall disease burden, as well as treatment response across multiple tumor types [40, 41, 42]. However, its sensitivity for intracranial disease remains limited.

This limitation may be due to the blood–brain barrier (BBB), which limits tumor DNA release from CNS lesions into the blood, reducing plasma ctDNA sensitivity for intracranial disease [43].

In contrast, CSF ctDNA positivity was associated with a 2.75‐fold increased risk of mortality (HR = 2.75), highlighting its strong prognostic value in patients with brain metastases. Compared with plasma, CSF is enriched with tumor‐derived DNA and more directly reflects genomic alterations arising from intracranial lesions. CSF ctDNA appears to be a more sensitive biomarker for evaluating CNS tumor dynamics [16, 44, 45, 46, 47].

Importantly, our findings delineate distinct yet complementary roles of plasma ctDNA and CSF ctDNA. Plasma ctDNA reflects systemic tumor burden and is suitable for early risk stratification and longitudinal monitoring. In contrast, CSF ctDNA more accurately captures intracranial genomic alterations and provides superior sensitivity for evaluating brain metastasis‐specific disease dynamics.

From a translational perspective, these two biomarkers can be integrated into a clinically applicable combined plasma and CSF ctDNA analysis. In clinical practice, plasma ctDNA may serve as a first‐line, minimally invasive tool for systemic surveillance and early identification of patients at high risk of metastasis or recurrence. For patients with suspected or confirmed CNS involvement, CSF ctDNA analysis can refine intracranial molecular characterization, guide targeted therapy, and enable real‐time monitoring of treatment response.

A multicenter prospective study demonstrated that ctDNA detection in CSF of glioma patients is more frequent and more concordant with tumor tissue than in other sample types, supporting CSF as the primary source for molecular characterization. In contrast, plasma ctDNA is detected less frequently, likely due to the BBB, but may still provide complementary information, particularly in cases of high tumor burden or barrier disruption [48]. Together, these findings support an integrated plasma and CSF ctDNA analysis for more comprehensive disease assessment. Such a stepwise clinical workflow has particular relevance in scenarios of “neurosystemic dissociation,” such as in HER2‐positive metastatic breast cancer, where extracranial disease may be controlled while CNS progression occurs. In these cases, integrated analysis of plasma and CSF ctDNA can provide a more comprehensive and dynamic assessment of disease status than conventional imaging alone, thereby facilitating timely therapeutic adjustments and avoiding ineffective treatments [45].

The lack of significant associations observed in melanoma and lung adenocarcinoma subgroups should be interpreted cautiously, as these findings may reflect limited sample sizes, tumor‐specific ctDNA shedding patterns, and variability in BBB permeability rather than a true absence of association.

Nevertheless, several limitations should be acknowledged. The relatively small number of included studies, heterogeneity in study design and detection methods, and variability across tumor types may limit the generalizability of the findings. Additionally, the observational nature of the included studies precludes causal inference. Furthermore, the integration of plasma and CSF ctDNA was not directly evaluated in prospective clinical trials. Future large‐scale, well‐designed studies with standardized protocols are warranted to validate these findings and to establish optimal clinical implementation strategies.

Taken together, our findings support using plasma and CSF ctDNA together to enhance early detection, prognostic assessment, and precision treatment planning for patients with brain metastases.

## 5 Conclusion

In conclusion, ctDNA represents a clinically meaningful biomarker for both risk prediction and prognostic assessment in brain metastases. Plasma ctDNA serves as a minimally invasive indicator of systemic tumor burden, whereas CSF ctDNA provides superior sensitivity for intracranial disease characterization. The integration of these biomarkers within an integrated plasma and CSF ctDNA analysis provides a clinically actionable strategy to improve early detection, refine prognostic stratification, and guide precision therapeutic decision‐making.

## Author Contributions

Shaobo Yang and Shun Yang conceived and designed the study. Hao Dong, Xin Yu, and Fangdi Zhu collected the data. Zongheng Zhang and Shaobo Yang performed the data analysis. Xin Yu, Fangdi Zhu, and Hao Dong prepared the figures and tables. Zongheng Zhang drafted the manuscript. Shaobo Yang and Shun Yang critically reviewed and revised the manuscript for important intellectual content. All authors participated in the discussion of the results and approved the final version of the manuscript.

## Ethics Statement

Ethical approval was not required for this study because it is a systematic review and meta‐analysis based on previously published data.

## Conflicts of Interest

The authors declare no conflicts of interest.

## Data Availability

The data that support the findings of this study are available from the corresponding author upon reasonable request.
